# A Practical Treatise on Enteric Fever; Its Diagnosis and Treatment

**Published:** 1860-01

**Authors:** 


ART. III.—*A Practical Treatise on Enteric Fever; its Diagnosis and Treatment; being an Analysis of One Hundred and Thirty Consecutive Cases, derived from Private Practice, and embracing a Partial History of the Disease in Virginia.* By JAMES E. REEVES, M D. Philadelphia: J. B. Lippincott & Co., 1859. 12mo., pp. 199.

THIS is one of a class of works of which we should like to see many more examples. It merits its qualifying epithet of "practical," without its being, what is too often regarded as the synonym, empirical. As exhibiting the results of extensive opportunities for observation of a disease which is becoming more and more prevalent, and which is at all times of doubtful, and, in a great many instances, of fatal termination, this treatise claims an attentive perusal. The circumstances under which it was prepared furnish an additional *a priori* argument in its favor, which is made the more plausible by the calm, clear, and modest style in which the subject is presented for our consideration. The author, in his preface, tells us, as a reason for his adding another work to the many previously written "on enteric fever," "that the material out of which this volume is compiled, has been collected in private practice, rural practice, and in a country where, on account of the frequency of the disease during the past fifteen years, extensive opportunities have been afforded for a thorough understanding of its symptoms, uncontrolled by the influences that operate in the production, march, and termination of the disease in city and hospital practice." Again: "This little volume, herewith submitted, is just as plain and simple a statement of an important disease as I could make. Its facts have been gathered at the bedside of the rich, and the *very* poor, in the well-situated and comfortable dwelling, and in the unfavorably-situated and poorly-constructed cabin-house, and among all ages." Regret is expressed by the author at the meagre contributions of country physicians to the store of published medical knowledge.

"Such is the extent of the apathy which prevails among the members of the medical calling, that our literature is entirely wrought from the labors of the few—professors of schools, and those in charge of hospitals. Were it not for the laudable endeavors of these men, we should not have an American medical literature."

With some noticeable exceptions, this statement is a correct one. The remark which follows, that "in many of the States the profession is represented by one or more well-conducted journals," hardly does justice to the amount and quality of the matter supplied to the common stock by works of this description. The use to which these periodicals are put in this way is evinced, among innumerable other instances, by an account of the cases of enteric fever reported by Dr. Reeves himself in 1856, for the Buffalo Medical Journal, and which constitutes the basis of the present treatise. There are various causes which prevent large contributions being made by country practitioners, such as want of time, and of habits of literary composition, and a doubt of their possessing the ability to communicate information that might interest and instruct their professional brethren. A little method would go far to obviate the difficulty alleged on the score of want of time. Few men, after a day of fatigue duty in traveling over the country and visiting the sick, are in a mood, when night comes, even if they are then left free from new calls, to take pen in hand and to prepare a paper for a journal, which demands much thought and reflection, and the active exercise of their reasoning powers. But every man in professional life is able to keep a daily record of his observations and experience, which shall be merely the expression of facts and phenomena occurring in the sick-room. From these materials he may, watching the genial hour or the quiet half day allowed to the most busy, at intervals, whether long or short, weave a historical or descriptive essay, which can hardly fail to be useful when spread on the pages of a journal. It is not necessary that a production of this nature should be marked by elegance of style, recondite research or learning. These are not requisite to attract the attention of his readers, the great majority of whom are in quest of positive information germain to their own wants, and such as shall either supply their deficiencies or confirm principles which they entertain themselves, but respecting which they may still have their doubts. In the records of the experience of a practicing physician we do not look for learning, and do not feel challenged to criticism unless when we see obtruded on us an ostentatious display of authorities of doubtful bearing on the subject in hand. A young physician may have met with cases in his practice of a somewhat unusual character, and he determines, in consequence, very properly, to give them publicity through the pages of a medical journal. But he is not content to send them forth alone in a plain familiar dress; he thinks it necessary to make them figure in an essay on the disease of which these cases are examples, and, instead of clothing them in a plain garb, he contrives to envelop them in patches of retrospective lore, bedizened with speculations and fancies which, he may flatter himself, will be called ingenious theory, until at last his original production, in the shape of cases, is as hard to be reached as an Egyptian mummy in its endless succession of cerements.

We must not imitate the practice of those members of another profession who "think that they shall be heard for their much speaking," and whose arguments are to be measured by the hour, and not by the standard of law and logic. Happy would it be if the sufferers under these inflictions were confined to the judge and the jury in courts at home; but the same diarrhœa of words shows itself in our State and National Legislature, to the detriment of the real business of the country. Some excuse might have been offered for the wordy speculations of a young medical writer at a time when he but imitated the course of the professor to whom, a few years previously, he had lent a willing ear in the lecture-room. With a reform in the practice of the teacher comes naturally enough an amendment in the literary conduct of the scholar; and now we may congratulate ourselves on a very marked improvement in the articles written for medical journals. They are less diffuse in argument, and abound more in facts pertinent to the theme; they are also composed in a better, because a less ambitious style.

Country physicians of mature age, and with long experience in the treatment of disease, convinced of the uselessness of theoretical discussions and averse to book-making by spinning out the materials in their possession, decline to appear as authors. They forget, however, or do not reflect, that a tiny volume may have more vitality and be more useful than an inflated quarto or double octavo. The instructive volume of Dr. Reeves is comparatively a small one, and it might, without injustice to himself or his subject, have been of a still more reduced bulk. The ever-celebrated treatise of Hippocrates on "Airs, Waters, and Places," if set up in the same type, would not occupy more than fifty pages of this volume. Heberden's Medical Commentaries, a work more praised than read, the result of the observations on diseases at the bedside of the sick, during half a century, do not take up more than a moderately-sized octavo of 350 pages. Cleghorn's classic treatise on the "Climate and Diseases of Minorca," makes a small duodecimo. In thus encouraging the practitioner to keep a record of his cases, and, after a suitable period, to arrange and digest them for publication, we do not mean to advocate needless publicity, nor, in praising brevity, would we cast even implied censure on the authors of systematic works or of those demanding historical research, who are of necessity required to enter largely and in detail into the examination of the multiplied topics which lie in their way.

The treatise of Dr. Reeves may be looked at under two aspects—first, as a description of what came under his own observation in reference to the disease on which he has written; secondly, as a monograph, containing a summary of the generally received views, blended with his own, of its pathology and treatment. In the first we find much to commend, and could wish for still more than we have received. In the second we feel less satisfaction, and are tempted to become critics. The inciting consideration in the mind of the author was, doubtless, to communicate to his professional brethren the results of his personal observations and reflections on a fever which is gradually extending itself over different parts of the State of Virginia as it is over the western and southwestern regions of the United States. Our first impressions of the manner in which he has executed the self-imposed task were conveyed in our opening remarks. It remains for us now to convey to our readers some idea of the scope and contents of Dr. Reeves's volume.

In the first chapter, under the head of "Preliminary Remarks," the author states at once his preference for the title of "enteric fever" as applicable to that disease which is more generally known under the name of typhoid fever. We cannot coincide with him in this opinion, although it is sustained by the authority of Dr. Wood, a letter from whom he quotes on the occasion. The enteric lesion, in the shape of the eruptive patches, affecting the glands of Peyer and the ilium, is not always present; and there is an enteric fever, acute dysentery, in which a very different part of the intestines is the chief seat of the lesion. The designation of typhoid is objected to by this gentleman, as implying a resemblance to typhus; whereas he regards the two diseases as wholly distinct, the one from the other. We are not called upon to discuss this subject now, after the tolerably full examination of it in the March number of this Journal for 1857, in our review of Dr. Bartlett's work on the "Fevers of the United States." It is a matter of regret that Louis had not been better acquainted with the histories of fevers given by different English writers. He would have found in Huxham, Hilary, and others, clear descriptions of his typhoid, under the head of Slow Nervous Fever, or more frequently of Typhus Mitior; and he might probably have been induced to retain the original name for that disease, of which he has the merit of detailing the anatomical lesions with a minuteness and precision unknown before. If we adopt, as for ourselves we are willing to do, the term typhoid, it is precisely because we believe in the strong family resemblance between typhus gravior, the typhus petechialis of the present day, and typhus mitior, the typhus abdominalis of continental pathologists. The suffix oid, from *eidos*, expresses reresemblance but not identity of cause and nature; as, for example, when Maelzel, in his ingenious mechanism, gave the name of androides to the minute automatic figures resembling men, which he marshaled and manœuvred at will, it was not pretended that they had vitality, volition, or any characteristic of living man. The prevailing features in both typhus and typhoid are there dependent upon disorder of the brain and nervous system generally. It was this disorder that procured for the fever the designation of typhus, and, assuredly, it is quite evident in the other, even though milder form of fever, to justify a corresponding term typhoid, if we refuse to say typhus mitior.

Dr. Reeves divides enteric fever into the simple or mild, the intermediate, and the malignant, including the fatal. He admits the difficulty, in some instances, of drawing the line between these divisions, which, after all, may serve better to designate stages of the fever, varying in their duration and intensity, than distinct forms. The duration of the mild form may not exceed nine days, or it may be protracted to twenty, thirty, forty, and even fifty days. The malignant, often the sequel of the milder fever, is, however, so in many instances from the beginning or a few days thereafter. Under the head of Symptomatology, the author describes the "prodromata," in which there is no uniformity. A table is given, showing the period of the formative stage in one hundred and thirty consecutive cases, in his practice. The fifth day, counting from the first to the eighth, gave the largest number, or 24 males and 15 females. Of the one hundred and thirty cases, sixty-four were of the simple or mild form,—the symptoms of which are described. Seldom was there rose-colored eruption absent at some period of this form of the disease. The transparent vesicular eruption, called sudamina, was, on the other hand, noticed in but a few cases. Delirium is not a constant symptom, although its occurrence in waking from sleep, or during the night, is not at all uncommon. Mention is then made of the turn which the disease takes, either to aggravation or amelioration of the symptoms. Thirty-two cases presented the exasperation of symptoms characteristic of the intermediate form. "During the night the delirium becomes more constant, though in these cases never so profound but that the presence of the physician, or some strange visitor, causes the mind to rest correctly. A marked increase of tympanitic distention and diarrhœa is now generally observed." The rose-colored eruption, "upon an average," was not found to occupy as much surface as in the preceding form. Sudamina, whenever found, always appeared at this time. Epistaxis, when it did occur, was always more profuse than in the milder form. Sudden change for the worse, after a marked amendment in every particular, is not uncommon in this form. Twenty-five cases, passing through the milder gradations, resulted in the malignant form. The author now completes the history of the intermediate form, in order to present us a continuous history of the disease from the mildest to the most severe. We shall not follow him in this examination, but content ourselves with selecting for notice the excessive tympanitic distention, the offensive, tar-like stools, the more frequent and profuse hemorrhages, diminished urinary discharge which has the color of dark lye, low and muttering delirium, carpology, "involuntary discharges of urine and fæces, and there arises from the patient's body the most sickening odor." Next are spoken of "those cases, occasionally occurring, in which a degree of malignancy is observed in the beginning or very soon thereafter. Nine cases assumed this cast immediately on the full development of the fever."

The symptoms are recapitulated in the following order: 1. Headache. 2. State of the Mind and Senses. 3. State of the Muscles. 4. State of the Pulse. 5. Cough and Bronchial Râles. 6. Diarrhœa. 7. Tympanites. 8. The Rose-colored Eruption. 9. Hemorrhage. 10. Tendency to the Formation of Eschars. "In a few instances the delirium is strictly monomaniacal." "Dullness of hearing is one of the most constant alterations in the functions of the senses observed in enteric fever." "The change of pulse most frequently observed is that of frequency combined with weakness, and this change is in nearly exact proportion to the severity of the disease. In the mild form it rarely exceeds 115, and in quite a number of instances falls short of these figures, and in a very few instances it falls below the actual standard." "Cough more or less almost invariably attends the disease. It may exist in the beginning, or not make its appearance until the latter part of the second week; most generally it sets in during the first week." *Diarrhœa.* "There is perhaps no one symptom belonging to enteric fever which, according to the degree of its manifestation, is so well proportioned to the gravity of the disease as diarrhœa. Even when it does not exist among the prodromata, the bowels show an increased susceptibility to the action of cathartic medicine, and by the exhibition of such medicine to purge off the supposed *bad cold*, the irritability of the bowels is increased, and diarrhœa brought on much earlier perhaps than it would have naturally occurred. In many instances, however, the discharges are liquid and frequent in the beginning without any assignable cause, and in such cases the disease soon assumes a grave form." "Along with the diarrhœa of either form, straining sometimes occurs, and where it exists, is particularly complained of when the patient is at stool." "Among the earliest symptoms of enteric fever is tympanites." "This symptom generally continues until the beginning of convalescence, or the termination of death." In Dr. Reeves's experience, he has seldom failed to observe the rose-colored eruptions at some stage of the disease.

The author begins the chapter on Anatomic Lesions by the remark: "If I were compelled to rely upon my own observation for a description of the changes found in the several organs and structures after death from enteric fever, I should omit this chapter." Forgetting the terms of his title-page, viz., of his treatise being an analysis of cases derived from private practice, he ceases here to be his own historian, and, desirous of making his work a monograph, he draws on another source to fill up the blank. He copies accordingly from Dr. Wood (*Treatise on the Practice of Medicine*) an account of the morbid anatomy of the disease. He could not, of course, have taken a safer guide.

Under the head of History and Causes, Dr. Reeves tells us, that "enteric fever has become a common disease in Virginia, and in some localities has almost completely supplanted the bilious-intermittent form, which was of such frequent occurrence in former years. This remark applies with especial truth to some sections of eastern and southeastern Virginia." The author introduces a number of letters from practitioners in that section of the State, to confirm this assertion. But on extending his view, he finds that the fever is common in the great valley as well as in the southwestern counties. His rapid notices of different districts and counties will only be understood by those familiar with the geography of Virginia, and even they will call a map into requisition to aid them in the search. The value of this part of the volume would be greatly enhanced by a minute topographical description of some of the localities in which the fever prevailed, as well as a specification of the situation and internal economy of the houses, and the race, (white or black,) ages, habits, and occupations, of those who were attacked by the disease. Details of this nature, always necessary, assume still greater importance when the question of contagion is brought up, as it is, in due course, by the author. To a limited extent, the details of a topographical nature now asked for, are given by Dr. Reeves in the instance of Philippi,—his place of residence, in Barbour County, and that in which the origin and progress of the fever shows, he thinks conclusively, its contagious character. The subject is of so much importance as to justify our giving the entire narrative, in the words of the author, as follows:—

"My own opinion is not wavering upon the subject, and I do not hesitate to declare my firm belief of its contagiousness. I have reserved the history of the disease in Barbour County until now, because of the evidence of contagion which it will exhibit.

"Enteric fever was not recognized in Barbour prior to the year 1837. In October of that year, a gentleman by the name of Selvey, who had been on a visit to Athens County, Ohio, was brought home sick of the disease, called at that time in Ohio, '*slow fever.*' In three or four weeks after his arrival, his mother, father, and three brothers were taken down with the disease; the mother died. The last of November following, six of another family near by, took the disease, and one died. In December it appeared in a third family; five of these had the disease, and three died. From these it sprang up in another family five had the disease, and one died. The last of January, 1838, nine of another family had the disease; four died. During the following spring and summer two other families took it; nine of these died. In the winter of 1838 and 1839, several other families took the disease, amounting in all to twenty cases; of these six died. From that time up to the winter of 1840 and 1841, there were occasional cases springing up throughout the country. But during the fall and winter of 1841, the disease appeared in another neighborhood, ten miles distant; several families were attacked; among which there occurred ten cases, and four died. In the fall of 1842, a merchant returned from the North, sick with the disease, (his neighborhood alike previously free;) several of his family took it, but none died. The following winter it appeared in another neighborhood; six had the disease, and three died. From this date up to the winter of 1843, there were but few cases occurring; but during the next spring it made its appearance in still another neighborhood; some ten cases occurred, and four died. During the year 1845 it was generally prevalent in the county; about thirty cases occurred, and but one or two deaths. From the close of the year 1845 up to the year 1851, the disease became less common; but during the spring and summer of the last-named year the extent of its visitation exceeded that of any former year; and the citizens of our village, particularly, suffered severely from the disease.

"Philippi is situated about ten miles west of the Laurel Mountain, on the Tygart's Valley River, forty miles from its junction with the Monongahela, which, for two miles above and five miles below, has a sluggish current. Across the river, immediately opposite Philippi, is a small village, Georgetown. The bank on that side is very low for a few hundred yards, spreading out into a level in extent of about ten acres, and bordered on both sides by high hills. The greater portion of this ground is inundated at every considerable rise of the river. In the spring of 1851 the construction of a bridge, connecting Philippi with Georgetown, was commenced; adding about thirty to the population of this last-named village. Among the workmen, whose business required them to stand in the water, enteric fever began; from these it occurred among the workmen in stone upon the banks, and from these to the citizens generally. Our side of the river, Philippi, was next attacked, extending from family to family. Several of the workmen at the bridge, on being taken sick, went to their families in various parts of the county, forming starting-points of the disease in their several neighborhoods. Other hands were employed to fill the places of those taken sick, but were soon likewise taken down. Fresh workmen were again and again secured; but the disease as successively continued to attack them, until at last, during the year 1852, the work was, in consequence of it, almost completely suspended.

"In September of this year, 1851, there was a menagerie exhibited at Philippi, which was largely attended by the people of the county; the following night nine persons, in attendance during the day, began to feel unwell, and had the disease.

"From 1851 to 1858, our county has never been entirely free from the disease for more than a few months at a time. During this period it has been wandering about the different neighborhoods, at one time mild, at another severe, attacking one neighborhood this year, another the next; and then, perhaps, returning to the locality it visited the year before. The fall and winter of 1855, the disease was uniformly severe. The summer was wet throughout; vegetation grew rapidly and was abundant. Heavy frosts appeared early, and the offensive exhalation from the decomposition of vegetable matter was remarkable. Hemorrhage from the bowels was of frequent occurrence at this season.

"I could bring forward other testimony of the contagiousness of enteric fever in abundance, but this I deem unnecessary, and shall content myself with the following single instance:—

"Riley M., aged twenty-four, a farmer, visited an acquaintance sick of the disease, October, 1855, distant some ten miles. There was no fever in Mr. M.'s neighborhood at the time of his visit to this friend, nor had there been for several years; but in two weeks after his return, he took the disease at his father's dwelling, and in twenty-eight days died. In ten or twelve days after his death, his sister, two brothers, mother and father, took it; the mother died. Among those who visited Mr. M., during his illness, was a cousin, a girl of nineteen, and a male friend, both of whom immediately contracted the disease; the cousin died. From these, other cases occurred in the neighborhood, no one taking the disease who had not visited the sick. Others were exposed also, but were not attacked.

"Dr. Elam D. Talbott, of Philippi, who has watched the progress of the disease in Barbour from the date of its advent, in 1837, to the present, is a firm believer in its contagiousness. This gentleman has met with the disease more often than any other of his county brethren; and though his usefulness is for the present denied, on account of the heavy hand of affliction which rests upon him, his long successful practice will be remembered by the community and acknowledged by his professional acquaintances."

Dr. Reeves, in common with all his professional brethren from whom he has made inquiries on this point, knows of no instance of a person having had a second attack of the fever. The experience of his correspondents is the same with that of Louis and others, that typhoid fever is most apt to make its attacks on persons in the prime of life; but it points to a wider range of age than was admitted by the French author. Dr. Reeves, in a table of 130 cases, which came under his own observation, shows that, among the males, he had a patient with this disease who was but eighteen months old, another thirty months, and seven from two to seven years of age. Cases of seven females furnished one at eighteen months, two from three to six years, and four from six to nine years. At the other end of the scale there were three male patients, whose ages were from fifty to sixty years, and one female at sixty to sixty-five years. Could not this person tell her age, and was the doctor left to guess it in this fashion? As relates to sex, the males furnished the majority of cases—a result which Dr. Reeves believes to be "entirely accidental."

The author, after quoting the observations of his brother practitioners in different parts of Virginia on the duration of the disease, gives the results furnished by his own experience. These show, that, in sixty-three cases of the mild form of enteric fever, the termination of the majority of them was between the thirteenth and eighteenth days. In eighteen cases of the malignant form the average duration was twenty-two days.

Touching the complications of enteric fever with other diseases, the earliest were found to be bilious-remittent fever and acute pneumonia; pleurisy in a few cases, and erysipelas in others, were met with during the progress of the fever. "The most fearful of the accidental inflammations is peritonitis, the result of perforation, and occurs sometimes when least expected." Bartlett is quoted for a description of the symptoms which accompany this accident.

The terminations and sequelæ of enteric fever are next noticed. Under the head of the first come convalescence and death. Among these latter Dr. Reeves has "seen two instances of swelled leg and thigh, like that which is incidental to parturient women." A well-written chapter on Diagnosis, Mortality, and Prognosis follows, closing with some remarks on "Nature." "Our powers at accurate diagnosis may sometimes be heavily taxed to distinguish enteric fever from puerperal peritonitis, especially when the symptoms of the latter bear the typhoidal relationship." As a general rule, the disease is believed by the author to be more fatal during the fall and winter months than at other seasons. In a preceding page, when speaking of enteric fever being long confounded with true typhus fever, he tells us that the doctrine of the identity of the two diseases is still adhered to, "notwithstanding the conclusive researches of several years, which have established beyond all cavil the dissimilarity of the two affections." So far from joining in this conclusion we would say, repeating the words of the author as applied to puerperal peritonitis, that our powers of accurate diagnosis may sometimes be heavily taxed to distinguish enteric fever from typhus fever. If the dissimilarity between the two be so evident, why have they been so repeatedly mistaken for each other? Huss, giving the result of twelve years experience of himself and his colleague, Professor Malmsten, on 3185 typhus patients, in the Seraphin Hospital in Stockholm, including under this head those who had suffered from typhus proper (*typhus petechialis*) and from typhoid fever, (*typhus abdominalis,*) uses the following language: that these fevers, "such as they appear in the climate of the North, neither can nor ought to be considered as two distinct maladies, but only as two varieties of the same morbid process." Elsewhere, using the words of philosophic doubt, he says: "It may be very easy to distinguish the two remotest links in the chain of which the typhus process may be said to be composed, and then to decide if any particular case is one of typhus or typhoid fever, but even the most penetrating discernment may be baffled in attempting to distinguish one of the intermediary forms, which lie between the two extremes, and partake a little of each, and it may be impossible to make a correct diagnosis."* Huxham, more than a century ago, expressed himself in nearly similar terms. When describing the two fevers, (the slow nervous and the putrid malignant,) he says that he is "very sensible the one may be, and very often is blended with the other." But, as we said before, we do not mean to renew the argument at this time, and what little we say on the subject is merely to show that Dr. Reeves is somewhat hasty in his sweeping assertion, that the dissimilarity of typhus fever and enteric or typhoid fever has been proved beyond cavil.

Sensible remarks are made on points of the prognosis of enteric fever, which we shall not specify, any more than details of the treatment of the disease. The latter is not exhibited as that which is pursued by the author, but rather as "a fair exposé of the subject." This we can get in many quarters. What would have been more acceptable and instructive, both in itself and for the purposes of comparison, was a specification of the measures coming within his own experience. To a certain extent this is done in his commentaries on the value set on the different remedies and classes of remedies by other writers and practitioners. In speaking of tepid sponging, of which he thinks favorably in the severer forms of the disease or when the patient is much prostrated, he makes the mistake, too common among practitioners and some medical writers, of confounding tepid and warm. To the former he assigns the limits of 87° to 97° Fahrenheit—a range which comes within a degree of the upward limit of the warm and the beginning of the hot bath, (98° F.)† The only new remedy in the treatment of enteric fever mentioned in the volume of Dr. Reeves is *veratrum viride.* Of the power of this drug to control the heart's action he is "as well convinced as of any other established truth in medicine." He speaks favorably of its diaphoretic property—a fact not generally noticed. The author regards it, in all proper cases of enteric fever, "as a safe, reliable and valuable adjuvant to other means employed for its manage-

* Statistics and Treatment of Typhus and Typhoid Fever, quoted in North American Medico-Chirurgical Review, p. 184, 1857.

† See Bell on Baths, etc.

ment." He has administered it to the young and to the aged, to the delicate and robust, in the pregnant state at all periods, and has had no cause to regret its effects. So far from its being apt to produce abortion, he thinks "further experience may possibly establish that, in certain conditions, the *veratrum viride* may be beneficially employed for the purpose of defending the pregnant state." A case is detailed in corroboration of this view. Dr. Reeves is willing to declare it a most valuable auxiliary, in many instances, to other recognized means, in moderating the violence of enteric fever, and in conducting it to a favorable termination. He judiciously adds: "I do not think that we are authorized to believe that the disease can be suddenly interrupted by any method of treatment with a view to its full effect." He usually begins by giving five or six drops of the saturated tincture in a little sweetened water, and repeats the dose every three hours, (increasing the quantity by two drops at each successive dose,) until the pulse is brought down to or below the natural standard. "Ordinarily it requires the fourth dose to effect complete mastery of the pulse." After "the telling dose" has been reached, the influence of the medicine may be prolonged by the administration of from two to eight drops every three or four hours. Among the good effects procured by this medicine, we learn that "the appetite was better sustained and the thirst less urgent than when not used." Next in the scale of novelty, is the chlorate of potassa in the treatment of enteric fever. Dr. Reeves has been in the habit of employing this agent in almost every case of the disease that has fallen under his care for the last five years. He directs it in weak solutions as a constant drink, when sordes begin to form about the lips, gums, and teeth. It is, he believes, an excellent vehicle for the administration of the veratrum viride, and as such may be used according to the formula proposed by Dr. Taliaferro. ℞.—Chlorat. Potass., saturated solution; Tinct. Verat. Virid., ℨss. M. Of this the patient is to take a tablespoonful every three hours during the day. Judicious advice is given in the use of stimulants and tonics. Opium is classed with the former: its combination with camphor, as a means of quieting nervous agitation and restlessness and of inducing sleep, is properly lauded.

Dietetic management and the treatment of complications next engage the notice of the reader. Strict attention to diet is not less important than the use of medicines in the successful management of the sick. Great watchfulness is very properly enjoined in the convalescences from enteric fever. The most important part refers to diet, regulation of the bowels, and exercise, on each of which good advice is offered. The following caution, too often unheeded or overlooked by medical men in prescribing a diffusible stimulant in depressed states of the system, is laid down by Dr. Reeves, viz.: "As health becomes confirmed, it should be gradually withdrawn." It ought to be an affair of conscience with every physician to keep watch over his patient until the use of a diffusible, by which we mean more especially an alcoholic stimulant, is entirely dispensed with.

